# Carbonic Anhydrase XII Expression Is Modulated during Epithelial Mesenchymal Transition and Regulated through Protein Kinase C Signaling

**DOI:** 10.3390/ijms21030715

**Published:** 2020-01-22

**Authors:** Daniele Vergara, Sara Ravaioli, Eugenio Fonzi, Loredaria Adamo, Marina Damato, Sara Bravaccini, Francesca Pirini, Antonio Gaballo, Raffaela Barbano, Barbara Pasculli, Julien Franck, Isabelle Fournier, Michel Salzet, Michele Maffia

**Affiliations:** 1 Department of Biological and Environmental Sciences and Technologies, University of Salento, 73100 Lecce, Italy; lorelai333@hotmail.it (L.A.); marina.damato@unisalento.it (M.D.); 2 Laboratory of Clinical Proteomics, “Giovanni Paolo II” Hospital, 73100 ASL-Lecce, Italy; 3 Istituto Scientifico Romagnolo per lo Studio e la Cura dei Tumori (IRST) IRCCS, 47014 Meldola, Italy; sara.ravaioli@irst.emr.it (S.R.); eugenio.fonzi@irst.emr.it (E.F.); sara.bravaccini@irst.emr.it (S.B.); francesca.pirini@irst.emr.it (F.P.); 4 CNR-NANOTEC, Institute of Nanotechnology c/o Campus Ecotekne, 73100 Lecce, Italy; antonio.gaballo@nanotec.cnr.it; 5 Fondazione IRCCS Casa Sollievo della Sofferenza Laboratorio di Oncologia, 71013 San Giovanni Rotondo, Italy; r.barbano@operapadrepio.it (R.B.); b.pasculli@operapadrepio.it (B.P.); 6 Laboratoire Protéomique, Réponse Inflammatoire et Spectrométrie de Masse (PRISM), Université de Lille, INSERM, U1192 F-59000 Lille, France; julien.franck@univ-lille.fr (J.F.); isabelle.fournier@univ-lille.fr (I.F.); michel.salzet@univ-lille.fr (M.S.)

**Keywords:** carbonic anhydrase, epithelial mesenchymal transition, breast cancer, proteomics, PKC, transport metabolon, metabolism

## Abstract

Members of the carbonic anhydrase family are functionally involved in the regulation of intracellular and extracellular pH in physiological and pathological conditions. Their expression is finely regulated to maintain a strict control on cellular homeostasis, and it is dependent on the activation of extracellular and intracellular signaling pathways. Combining RNA sequencing (RNA-seq), NanoString, and bioinformatics data, we demonstrated that the expression of carbonic anhydrase 12 (CAXII) is significantly different in luminal and triple negative breast cancer (BC) models and patients, and is associated with the activation of an epithelial mesenchymal transition (EMT) program. In BC models, the phorbol ester 12-myristate 13-acetate (PMA)-mediated activation of protein kinase C (PKC) induced a down-regulation of CAXII with a concomitant modulation of other members of the transport metabolon, including CAIX and the sodium bicarbonate cotransporter 3 (NBCn1). This is associated with a remodeling of tumor glycolytic metabolism induced after PKC activation. Overall, this analysis highlights the dynamic nature of transport metabolom and identifies signaling pathways finely regulating this plasticity.

## 1. Introduction

Epithelial cells finely control the transport of ions, water, and other molecules inside and outside the cell under different physiological and pathological conditions. This is obtained through the expression of a plethora of channels, and transporters that physically and functionally interact with other membrane-associated proteins, to generate the so-called “transport metabolon” [1]. Modifications of this cellular architecture through the activation of processes of cellular transformation including the epithelial mesenchymal transition (EMT) have profound effects on the expression of transport metabolon proteins [2,3].

Carbonic anhydrases (CAs) are ubiquitous enzymes that catalyze the interconversion between carbon dioxide and bicarbonate thus regulating intracellular and extracellular pH and volume. For instance, in cells with high glycolytic metabolism, the CAII colocalizes with the monocarboxylate transporter MCT1 to facilitate transport activity [4]. Thus, modifications in the expression of CA isoenzymes may have profound effects on cell physiology. This is based on in vitro, ex-vivo, and clinical data [5,6,7,8,9,10]. The CAIX is one of the best-studied members of this family [6]. More recently, the role of CAXII has come into the spotlight due to its potential clinical relevance. A key concept emerging from these articles is the unique expression of CAIX and CAXII among breast cancer (BC) subtypes [7]. CAXII expression was confirmed by immunohistochemistry in a cohort of 103 breast tumor samples and associated with lower tumor grade, positive estrogen receptor (ER) status, negative EGFR status, and the absence of necrosis [8]. In vitro, the exposure of BC cells to estradiol (E2) significantly up-regulates *CA12* gene expression through a hormone-responsive enhancer located about 6 kb upstream of the start site of transcription of the *CA12* gene [9].

In our precedent study, we focused on exploring the biological differences between epithelial and mesenchymal BC cell lines by using mass spectrometry techniques [3]. This approach classified differentially expressed proteins into enriched networks and pathways associated with distinct EMT phenotypes [3]. We also focused on providing functional data about single proteins that play a prominent role in EMT initiation [10]. From these analyses, changes in the expression of metabolic enzymes and membrane transporters emerged as distinctive features of EMT. In this context, the possible correlation between CAXII and the EMT is still unclear. Here, we searched for this correlation by analyzing the expression of CAXII in BC human tissues and cell lines. CAXII expression is associated with the epithelial phenotype, and controlled by key regulators of EMT transition including transcriptional factors and protein kinases, including protein kinase C (PKC). Interestingly, the activation of PKC significantly reduced CAXII expression thus regulating in a coordinated manner the expression of other members of the transport metabolon.

## 2. Results

**Figure 1:**
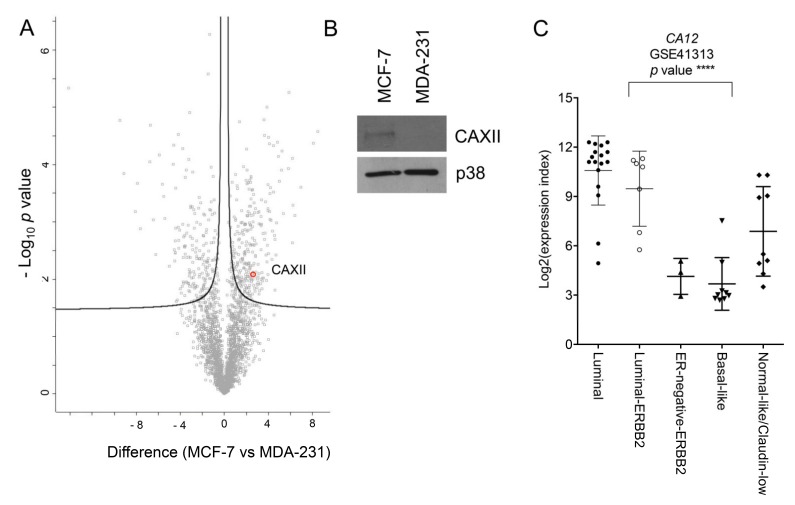
*CA12* is differentially expressed in breast cancer cell lines. (**A**) Proteins obtained by MS/MS analysis of MCF-7 and MDA-231 cells are ranked in a volcano plot according to their statistical *p*-value (*y*-axis) and their difference (log2 fold change). Carbonic anhydrase 12 (CAXII) is highlighted. The curve is derived at false discovery rate (FDR) = 0.05 and s0 = 0.1. (**B**) Western blot analysis of CAXII expression in MCF-7 and MDA-231 cells. P38 was used as a loading control. (**C**) Analysis of mRNA expression in breast cancer cell lines was performed using GEO dataset. Scatter dot plots show *CA12* expression levels in breast cancer cells from the GEO dataset GSE41313. *p*-value **** < 0.0001.

We explored the possible association between the EMT program and expression of CAXII in BC cell lines and in a cohort of 12 BC formalin-fixed paraffin-embedded (FFPE) samples. To do this, we first interrogated our proteomic data generated by the mass spectrometry (MS) analysis of the luminal model MCF-7 (estrogen-receptor (ER) and progesterone-receptor (PR) positive) and the triple-negative BC (TNBC) model MDA-231 (ER, PR, human epidermal receptor growth factor 2 (HER2)-negative) [3]. In this dataset, we observed an up-regulation of CAXII protein in MCF-7 cells compared to MDA-231 cells (Figure 1A). We confirmed this result by evaluating the expression of CAXII by western blotting. As shown, high levels of CAXII were observed in MCF-7 cells, whereas the expression was undetectable in MDA-231 cells (Figure 1B). In order to further support this data, we investigated *CA12* expression using microarray data retrieved from the Gene Expression Omnibus (GEO) dataset GSE41313 that includes a cohort of molecularly well-characterized BC cell lines. The analysis of this dataset confirmed that *CA12* is differentially expressed between luminal and basal-like models of BC (Figure 1C).

**Figure 2:**
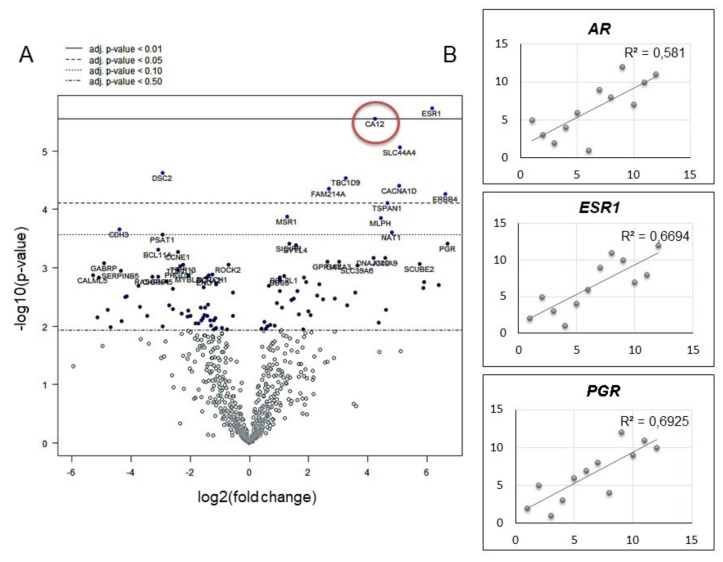
mRNA analysis of *CA12* expression in breast cancer tumors. (**A**) Differential expression between luminal A and triple negative patients analyzed on NanoString data: fold change of 4.23 (95%CI: 3.51–4.95, *p* = 0.0066). (**B**) Correlation of transcripts per million (TPM) counts analyzed by RNA-seq between *CA12* and hormone receptor genes: *CA12* showed a positive correlation with androgen receptor (*AR*), estrogen receptor (*ESR1*), and progesterone receptor (*PGR*) with a Spearman correlation coefficient of 0.766, 0.818, and 0.832, respectively. All coefficients were statistically significant (*p* = 0.0020; *p* = 0.00057; *p* = 0.00039).

Furthermore, we analyzed by using both NanoString and RNA-seq technologies the differential gene expression profile of a cohort of patients composed of 6 luminal and 6 TNBC tumor samples, whose subtypes were defined by an immunohistochemical (IHC) classification (Table 1). The differential expression of *CA12* was analyzed on RNA-seq data between luminal and TNBC. *CA12* was up-regulated in all luminal BC analyzed cases (log2 fold change 4.08, *p* < 0.0001), even considering only the luminal A patients (log2 fold change 3.94, *p* < 0.0001). Similarly, the analysis of NanoString gene expression profile data revealed a significant up-regulation of *CA12* in luminal A BC patients (Figure 2A). Among luminal B cancers, data analysis also demonstrated higher levels of *CA12* (log2 fold change 4.04, 95%CI: 3.31–4.76, *p* = 0.02).

We next assessed the correlation between *CA12* and hormone receptor genes (*AR*, *ESR1*, *PGR*) by using both RNA-seq and NanoString counts. Data from RNA-seq (Figure 2B) and NanoString counts (data not shown), demonstrated a strong positive correlation between *CA12* expression and hormone receptor genes (Figure 2B). These results were in line with those obtained analyzing the correlation between *CA12* NanoString normalized counts and ER and PR IHC score (% of immunopositive tumor cells) (Supplementary Figure S1). Pearson’s correlation coefficient between NanoString and RNA-seq counts was 0.72 (*p* < 0.0001). Out of the 734 genes shared by both methods, those differentially expressed between luminal and TNBCs were 135 by RNA-seq, 155 by NanoString and 88 according to both, including *CA12* gene.

**Figure 3:**
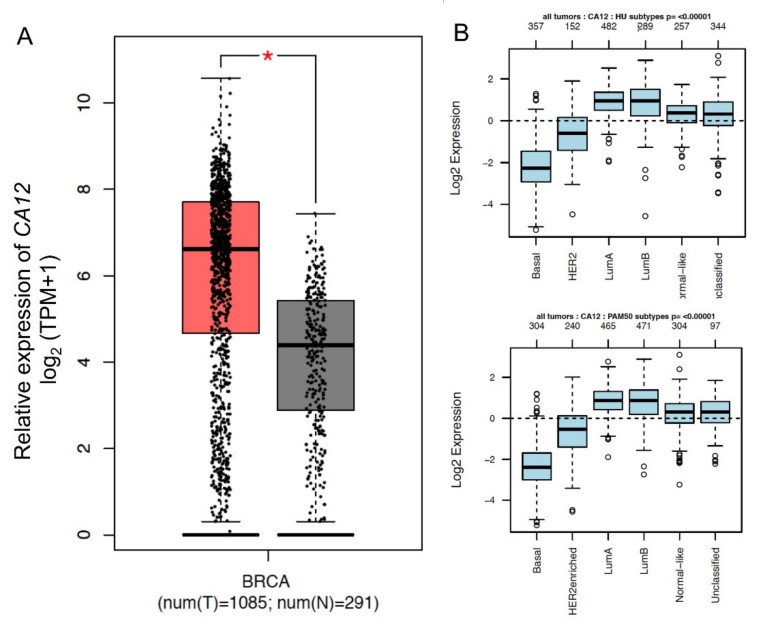
Bioinformatics analysis of *CA12* expression in breast cancer tumors. (**A**) Expression level of *CA12* in cancer and normal tissues. The RNA-seq data are expressed as relative gene expression using transformed log2 (TPM+1) value (Y-axis) of tumor (red) and normal (dark grey) samples from BRCA cancer types and displayed as a whisker plot. BRCA: Breast Invasive Carcinoma. *p*-value * < 0.01 based on one-way ANOVA. (**B**) *CA12* expression in six subtypes of breast cancer tumors. Patients were stratified according to HU and PAM50 subtypes applying the gene Set Analysis (GSA) of GOBO database.

**Figure 4:**
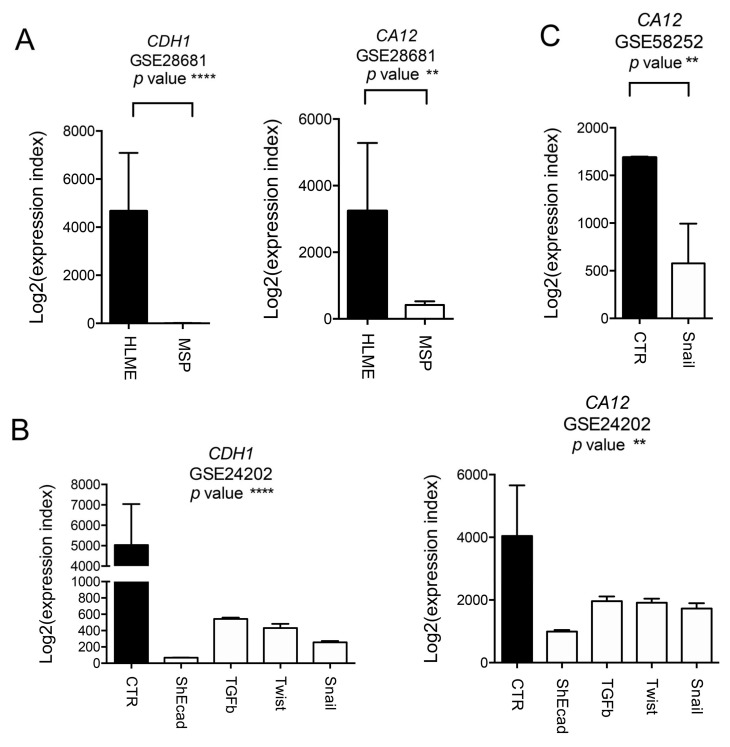
*CA12* regulation during epithelial mesenchymal transition (EMT). (**A**) Analysis of mRNA expression in breast cancer cell lines was performed using GEO dataset. Box plots show *CDH1* and *CA12* expression levels in the mesenchymal subpopulation (MSP) of cells isolated from immortalized human mammary (HMLE) cells. Data were obtained from the GEO dataset GSE28681. The *p*-value for the comparison between the two groups was determined using the Student’s *t*-test. *p*-value ** < 0.01, *p*-value **** < 0.0001. (**B**) Analysis of mRNA expression in breast cancer cell lines was performed using GEO dataset. Box plots show *CDH1* and *CA12* expression levels in immortalized HMLE breast epithelial cells retrovirally transduced in culture with vectors encoding EMT-inducing genes. Data were obtained from the GEO dataset GSE24202. The *p*-value for the comparison between different groups was determined using the ANOVA test. *p*-value ** < 0.01, **** < 0.0001. (**C**) Analysis of mRNA expression in breast cancer cell lines was performed using GEO dataset. Box plots show *CA12* expression levels in MCF-7 control (CTR) cells and MCF-7 cells over-expressing Slug. Data were obtained from the GEO dataset GSE58252. The *p*-value for the comparison between the two groups was determined using the Student’s t-test. *p*-value ** < 0.01.

**Figure 5:**
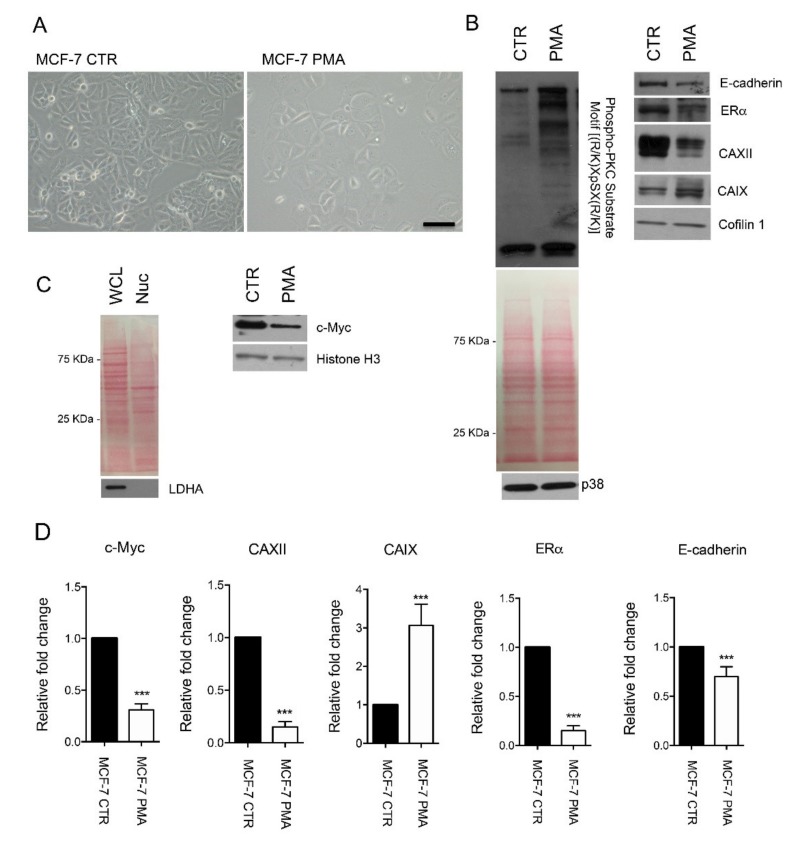
Protein kinase C (PKC) activation in MCF-7 cells induces changes in the expression of EMT markers and CAXII. (**A**) Representative images of MCF-7 cells treated with vehicle (DMSO) or phorbol ester 12-myristate 13-acetate (PMA) at the concentration of 100 nM for 48 h. Images were acquired using an inverted wide-field microscope (Olympus IX51). Scale bar 100 uM. (**B**) Cells were then harvested, and cell lysates were subjected to western blot using anti-phospho-PKC substrate motif [(R/K)XpSX(R/K)], anti-E-cadherin, anti-ERα, anti-CAIX, anti-CAXII antibodies. Anti-Cofilin 1 and anti-p38 were used as loading controls. (**C**) Whole cell lysate (WCL) and nuclear (Nuc) proteins were separated by SDS-PAGE and transferred to nitrocellulose membrane. Blots stained for WCL and Nuc proteins with Ponceau S are showed. Western blot showing L-lactate dehydrogenase A chain (LDHA) expression in WCL and Nuc fractions was used as cytosolic marker. Western blot analysis of c-Myc expression in the Nuc fraction of MCF-7 treated or not with PMA. Anti-Histone 3 was used as loading control. (**D**) Band density was measured using ImageJ. *p*-value *** < 0.001 by *t*-test.

Data extracted from the Cancer Genome Atlas (TCGA) database revealed that *CA12* expression is significantly higher in tumor samples compared to normal tissues (Figure 3A). We also determined its expression in relation to BC molecular subtypes by using GOBO database. We interrogated the software for *CA12* expression in breast tumor samples, and we observed a reduced expression of *CA12* in basal tumors compared to the other subtypes (Figure 3B). Overall, experimental and bioinformatics data supported the notion that *CA12* expression is increased in tumor samples but inversely associated with a more aggressive phenotype. To determine whether the shift between epithelial and mesenchymal markers that characterizes luminal and basal tumors can modulate *CA12* expression, we analyzed publically available GEO datasets in which EMT activation was obtained in breast models by the forced expression of EMT-transcription factors (TFs) or by extracellular stimuli (Figure 4). In the spontaneously arising mesenchymal subpopulation (MSP) of cells isolated from immortalized mesenchymal human mammary epithelial (HLME) cells or in HLME variants, in which the expression of Twist, Snail, the knock-down of E-cadherin, or the stimulus with TGFB was used to drive EMT, we observed that the reduced expression of *CA12* coincides with the decrease in the E-cadherin (*CDH1*) level and the acquisition of a mesenchymal phenotype (Figure 4A,B). Moreover, MCF-7 cells that over-expressed Snail also showed a decreased in *CA12* levels (Figure 4C). Overall, these data demonstrate that EMT activation leads to *CA12* down-regulation. To further investigate whether activation of signaling pathways involved in the acquisition of an EMT signature may modulate *CA12* expression, we stimulated MCF-7 with phorbol ester 12-myristate 13-acetate (PMA) to induce the activation of protein kinase C (PKC). Previous studies have shown that PKC may act as a positive regulator of EMT in BC [11]. In MCF-7 cells, PMA treatment induced morphological changes as visualized by inverted microscopy, a sustained activation of PKC as demonstrated by the analysis of phospho-PKC activated-substrates after 48 h of stimulation, and a down-regulation of the epithelial marker E-cadherin (Figure 5A,B), thus representing a trigger for the EMT program. Importantly, this was also accompanied by down-regulation of CAXII (Figure 5B). To investigate the role of ERα in the regulation of CAXII after PKC activation, we determined ERα levels by western blot. In support to the association observed by RNA-seq and bioinformatics, we observed significant reduced ERα levels in PMA-treated MCF-7 cells, indicating a possible effect on target genes controlled by ERα. To confirm this hypothesis, we validated the expression changes of the selected ERα target gene product c-Myc using western blot [12]. In the nuclear fraction of PMA-treated MCF-7 cells, we observed reduced levels of c-Myc compared to CTR cells, thus suggesting a reduced transcriptional activity of ERα (Figure 5C).

Taken together, these data show that PKC activation induces CAXII down-regulation by repressing ERα expression. As cells rely on CAXII expression to regulate extracellular pH, we believe that its down-regulation might require changes in the expression of the other membrane enzyme CAIX to support any cellular acid-base modifications. As shown, we confirmed that CAIX undergoes a significant up-regulation after PMA-treatment using western blot analysis (Figure 5B).

**Figure 6:**
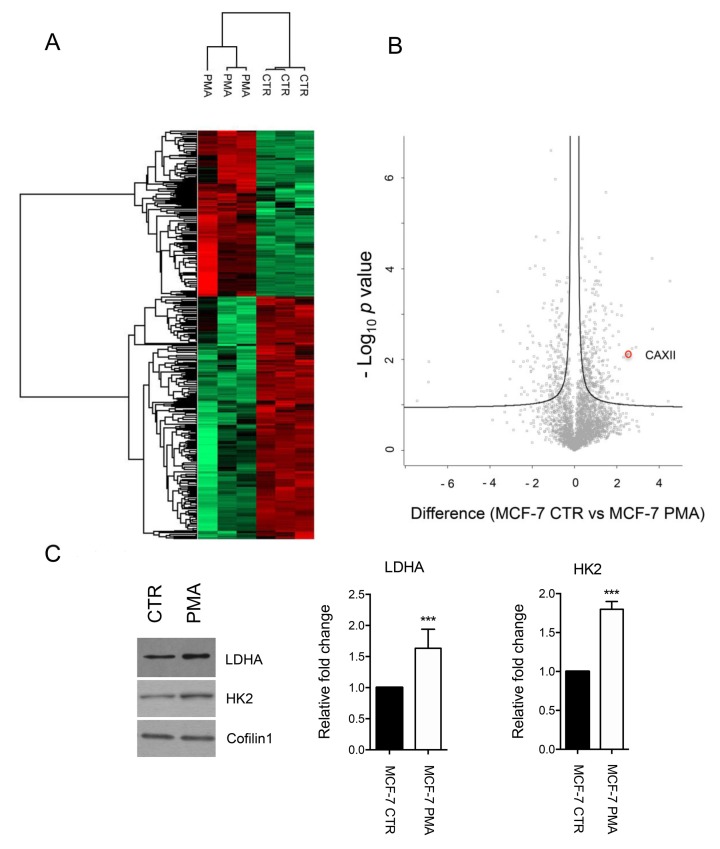
Mass spectrometry analysis of PMA-treated MCF-7 cells. (**A**) Heat map based on Euclidean distance that showed a significant separation between the control and PMA-treated MCF-7 cells. Color scale ranges from red to green (highest to lowest relative expression). Each column of the heat map represents an independent sample and each row represents a specific protein. (**B**) Differentially expressed proteins obtained by MS/MS analysis are ranked in a volcano plot according to their statistical *p*-value (*y*-axis) and their difference (log2 fold change). CAXII is highlighted. The curve is derived at FDR = 0.05 and s0 = 0.1. (**C**) Western blot analysis and band density quantification of LDHA and hexokinase-2 (HK2) expression in control and PMA-treated MCF-7 cells. Anti-Cofilin 1 was used as loading control. Band density was measured using ImageJ. *p*-value *** < 0.001 by t-test.

Having established the effect of PMA on the expression of CAXII, we subsequently determined proteome alterations induced after PKC activation. This unbiased approach might reveal modifications in other members of the “transport metabolom” thus providing further insights into the functional effects of PKC activation in epithelial cells. To do this, we used mass spectrometry to reveal proteome modifications induced by PKC activation in MCF-7 cells. Samples were digested with the proteolytic enzyme trypsin for subsequent proteomic analysis by liquid chromatography-mass spectrometry (LC-MS/MS). A total of 515 unique proteins showed significantly altered levels of expression in MCF-7 and PMA-treated cells (Supplementary Table S1A,B). Expression profiles of the identified proteins in MCF-7 samples are shown in Figure 6. This analysis clearly distinguished two main clusters thus showing that the proteomic profile of MCF-7 is significantly altered after PMA treatment. Gene Ontology (GO) enrichment analysis with respect to Kyoto Encyclopedia of Genes and Genomes (KEGG) pathways revealed a statistical enrichment of regulation of actin cytoskeleton (false discovery rate (FDR) 0.00042), focal adhesion (FDR 0.00042), and tight junction (FDR 0.0011) terms in MCF-7 cells after PMA treatment. In this cluster, proteins involved in the regulation of cytoskeletal dynamics and cell metabolism were also identified. Prominent examples are 14-3-3 proteins including YWHAZ, YWHAE, and YWHAB (Supplementary Table S1). In contrast, KEGG terms spliceosome (FDR 1.43 × 10^−19^) and DNA replication (FDR 1.72 × 10^−12^) were statistically represented among proteins increased in MCF-7 CTR. This is in agreement with the down-regulation of genes related to DNA synthesis and RNA processing that have been similarly observed in PMA-treated MCF-7 by microarray [13]. Considering other proteins significantly changed in MCF-7 cells and in line with western blot data shown in Figure 5, we observed the down-regulation of CAXII, and the up-regulation of transporters involved in the regulation of ion fluxes through plasma membrane, including the sodium bicarbonate cotransporter 3 (SLC4A7, NBCe1), the sodium/potassium-transporting ATPase subunit beta-1 (ATP1B1), and ATP1A1. Finally, PMA treatment was also associated with an increased expression of proteins involved in glycolysis including L-lactate dehydrogenase A chain (LDHA) and hexokinase-2 (HK2), as demonstrated by MS/MS and western blot (Supplementary Table S1 and Figure 6C). This supports the possible functional interplay between metabolic enzymes and transporters in mediating PKC cellular effects.

## 3. Discussion

Acidosis and hypoxia may be considered as two hallmarks of cancer and correlated with increased metastatic capacity and poor prognosis of multiple cancer types [14]. The mechanisms by which cells respond to these alterations of the microenvironment are finely regulated by a complex network of transporters and enzymes. CAXII and CAIX contribute to the maintenance of extracellular pH regulation and they are potentially involved in the other cellular processes including cell migration and stemness potential [15,16]. Other studies have also provided direct evidence of their role as potential clinical biomarkers. In primary breast tumors, CAXII expression was revealed by IHC in tumor cells, luminal cells of normal ductal epithelium and associated with the positive ER status. For CAIX, the expression was observed in stromal fibroblast and tumor cells. Moreover, CAIX staining in stromal cells was significantly associated with sentinel lymph node metastasis in addition to lymphatic invasion and a low Ki-67 labeling index [17].

Here, by using a combination of bioinformatics and experimental approaches we demonstrated that CAXII is up-regulated in BC samples compared to normal tissues, and expressed at highest levels in luminal subtype compared to triple-negative subtype. This was confirmed in BC cells by MS/MS and western blot and in human BC patient cohorts by RNA-seq. In this cohort, *CA12* expression is significantly correlated with the expression of *ESR1*, *PGR*, and *AR* thus suggesting that the signaling mediated by these receptors can have a role in the transcriptional control of *CA12* gene. While the activation of ERα by estrogen stimulation promotes *CA12* expression in BC models [9], the functional role of PR and AR remains unclear. The transcriptional cross-talk between AR, PR, and ERα is well established [18,19] and this may explain for the observed statistical association.

Multiple studies have demonstrated a correlation between ERα expression and the EMT program activation. In BC models, a down-regulation of ERα was observed after the activation of the EMT-TFs Snail and Zeb1 [20,21]. According to this, it can be speculated that modulation of the EMT status of epithelial cells may impact on *CA12* expression. This was demonstrated by the bioinformatics analysis of GEO datasets as shown in Figure 4. Interestingly, we observed a clear relationship between EMT state and *CA12* expression. Overall, this identifies *CA12* as a lineage epithelial marker and opens to the better comprehension of signaling pathways that may affect the expression of this enzyme by modifying the differentiation status of epithelial cells. In addition, in consideration of the strict regulation between *CA12* and the other members of the transport metabolom, this will permit to define the functional modifications that occur during all the biological processes that involve the loss of differentiation including EMT. To investigate this, we treated MCF-7 cells with PMA to induce the activation of PKC, a kinase involved in the activation of EMT. By inducing morphological modifications, and targeting the expression of the epithelial marker E-cadherin, PKC activation has a broad impact on epithelial cell identity. This is accompanied by a reduction of ERα expression and a significant down-regulation of the c-Myc TF. According to GO classification, focal adhesions and cell cycle proteins were significantly modulated after PMA treatment. We speculate that reduced levels of c-Myc may account for these changes. In fact, c-Myc is a positive regulator of cell cycle proteins [22], but also a suppressor of cell motility by targeting integrins [23]. In the work of Liu and collaborators, c-Myc depletion in MCF-7 cells significantly up-regulated the expression αv and β_3_ integrin subunits [23], as also observed in our proteomic dataset. It is still not clear if c-Myc may have a direct or indirect role in the regulation on *CA12* through an ERα-independent mechanism. In breast and cervix carcinoma cell lines, c-Myc depletion led to a down-regulation of *CA12* but not *ESR1* [24]. In addition, Hong and collaborators found a rare population of pre-adapted cells, isolated by MCF-7 treated with acute estrogen deprivation, which displayed features of mixed epithelial and mesenchymal traits, along with up-regulation of p53 and hypoxia pathway, with a reduced ERα activity and down-regulation of the cell cycle machinery and c-Myc target genes [25]. Thus, the possible ERα, c-Myc, CAXII functional axis warrants further investigation.

**Figure 7:**
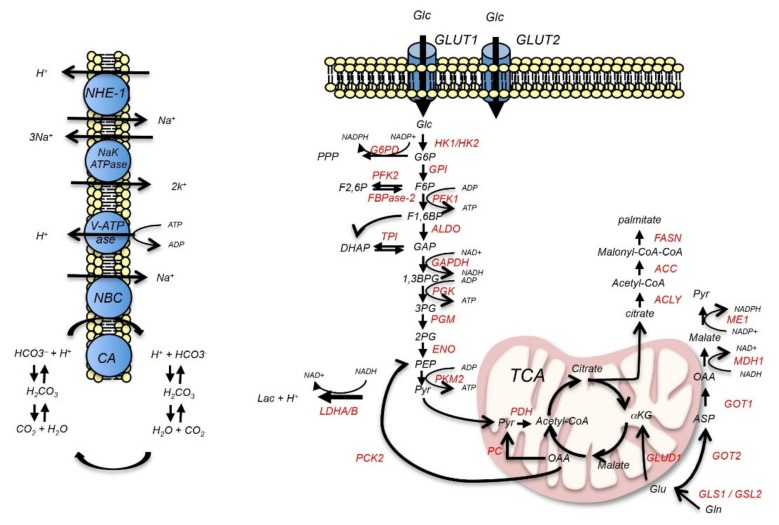
Schematic illustration of the effects of PKC activation on metabolic and transport metabolon proteins. After PMA-stimulation, PKC induces the up-regulation of LDHA and HK2. This is associated with an increased expression of sodium bicarbonate cotransporter 3 (NBCn1). Here, we also report that the expression of CAXII is down-regulated through a signaling that involves ERα. Modified from [3].

It is interesting to notice that, in reply to the down-regulation of CAXII, we observed an increase in the levels of CAIX. This finding can imply that a coordinated regulation between the two enzymes may take place to regulate the perturbations of extracellular and intracellular pH due to the loss of CAXII and other metabolic effects mediated by PKC activation. In fact, the unbiased proteomics analysis of MCF-7 treated with PMA revealed a metabolic rewiring toward a glycolytic phenotype together with an up-regulation of the sodium bicarbonate cotransporter 3 and two subunits of the sodium/potassium-transporting ATPase. These data suggest a model in which protons produced by the conversion of pyruvate to lactate due to the increased expression of LDHA are neutralized by the reaction with intracellular bicarbonate ions, generated by the hydration of CO_2_ by CAIX (Figure 7). This suggests that changes in the epithelial status induced by activation of EMT signaling pathways have profound effects on enzymes and transporters that regulate pH homeostasis with several functional implications. In breast cancer cell lines, NBCe1 supports cell migration, proliferation, and tumor growth through the regulation of intracellular pH [26]. Overall, these data demonstrate the usefulness of our proteomic data and their potential for future investigation into PKC signaling.

In summary, CAXII expression is correlated to the epithelial status of breast cells and modulated through the activation of specific signaling pathways. This is of potential clinical relevance. CAXII levels in breast cancer tissues are correlated with a better prognosis thus supporting its role as biomarker at the tissue level [8], while no studies have addressed the role of CAXII in other clinical samples including serum or plasma. As CAXII respond to the activation of estrogen signaling, the detection of CAXII levels in fluids can make it possible to correlate changes in this protein with changes of ER signaling pathway at the tissue level. Reinforcing this hypothesis, data extracted from the Human Protein Atlas (https://www.proteinatlas.org/) revealed that this protein is expressed in human plasma samples at the concentration of 29 ng/L, as detected by mass spectrometry. The strength of this hypothesis remains to be validated.

## 4. Materials and Methods

### 4.1. Human Tumor Samples and Gene Expression Profile

The 12 FFPE tumor samples were selected from a larger case series of BC patients enrolled in a retrospective study, carried out at IRST-IRCCS, in collaboration with the Cancer Prevention Unit and the Breast Surgery Unit of Morgagni-Pierantoni Hospital in Forlì. Eligible patients were aged ≥18 years with a histological diagnosis of invasive BC. The initial study protocol was reviewed and approved by the IRST and Area Vasta Romagna (AVR) Ethics Committee (approval no. 3692, 18 July 2014) and patients provided written informed consent. In order to select the most representative inclusion of tumor tissue for each patient, a pathologist reviewed the original hematoxylin and eosin stained sections. Tumors were classified according to the most recent St. Gallen classification. RNA was isolated from FFPE tumor with AllPrep DNA/RNA FFPE Kit (Qiagen, Hilden, Germany) and quantified by Nanodrop. The RNA quality was checked before performing NanoString BC 360^™^ panel assay (NanoString Technologies, Seattle, WA, USA) and preparing RNA-seq library (NEBNext Ultra II RNA Library Prep Kit, Illumina, San Diego, CA, USA) following the manufacturers’ instructions. Libraries were sequenced on NextSeq 500 (Illumina, San Diego, CA, USA). Reads were aligned using Kallisto and raw read counts were normalized as scaled reads per base [27].

### 4.2. Cell Lines and Culture Treatments

Human tumor cells were purchased from the American Type Culture Collection (ATCC, Manassas, VA, USA) or from Banca Biologica and Cell Factory (IRCCS Azienda Ospedaliera Universitaria San Martino-IST Istituto. Nazionale per la ricerca sul cancro, Genova, Italy). The human cancer cell lines MCF-7 and MDA-231 were cultured in DMEM medium (4500 mg/L glucose, EuroClone, Milan, Italy) supplemented with 10% Fetal Bovine Serum (FBS), 100 U/mL penicillin, and 100 μg/mL streptomycin at 37 °C in an atmosphere of 5% CO_2_. To induce PKC activation, MCF-7 cells were daily stimulated with phorbol-12-myristate-13-acetate (Santa Cruz) at the concentration of 100 nM for 48 h.

### 4.3. Sample Preparation, Mass Spectrometry Analysis, and Database Searching

Whole protein extraction was carried out with the Illustra TriplePrep kit (GE Healthcare, Chicago, IL, USA) according to the manufacturer’s protocol. Subsequently, proteins were processed according to the 10 K filter-aided sample preparation (FASP) protocol, as described [3]. Protein digestion was carried out at 37 °C overnight, peptides were desalted with C18 StageTips (Thermo Fisher, Waltham, MA, USA) and resuspended in 20 µL of ACN/H_2_O (FA 0.1%) (2:98, *v*/*v*). Peptides were then analyzed by high-performance liquid chromatography and analyzed by mass spectrometer on an Orbitrap Q-Exactive (Thermo Fisher, Waltham, MA, USA), as described [3]. Raw files obtained from MS were processed using the MaxQuant proteomic software (version 1.6.1.0) [28]. Q-Exactive spectra were matched to peptide sequences in the human UniProt protein database (release uniprot-human-reviewed-042018-20303seq.fasta) using the Andromeda algorithm [29]. Label-free quantification of proteins was conducted using the MaxLFQ algorithm [30]. Statistical analysis was performed with the Perseus software (version 1.6.2.1). Functional annotation and characterization of identified proteins were performed using STRING version 11 (https://string-db.org) [31].

### 4.4. Western Blotting

Cell lysates were extracted in RIPA buffer (Cell Signaling) and protein concentration was determined by the Bradford protein assay (BIORAD, Hercules, CA, USA). Nuclei were isolated using the Nuclei EZ Prep Kit Nuclei Isolation Kit (SIGMA, St. Louis, MO, USA) and lysed in RIPA. Samples were mixed 1:1 with Laemli buffer (SIGMA, St. Louis, MO, USA), boiled for 5 min and 25–40 μg of proteins were separated by 12% SDS–PAGE and transferred to the Hybond ECL nitrocellulose membrane (GE Healthcare, Chicago, IL, USA). The membranes were blocked for 1 h in Blotto A (Santa Cruz, CA, USA) at room temperature and subsequently probed by the appropriately diluted primary antibodies for 1–2 h at room temperature. After three washes with a solution containing 10 mM Tris, pH 8.0, 150 mM NaCl, 0.5% Tween 20 (TBST solution), blots were incubated with secondary antibody HRP-conjugated for 2 h at room temperature (1:2000 dilution). Blots were then developed using the Amersham ECL western blotting detection system (GE Healthcare, Chicago, IL, USA). Images shown in the paper are representative of at least three independent replicates.

Primary antibodies (1:1000 dilution) were: from Atlas Antibodies, CAXII (AMAb90639); from Santa Cruz Biotechnology, ERα (sc-7207), CAIX (sc-365900), LDHA (sc-137243), HK2 (sc-374091); from Cell Signaling, Phospho-PKC Substrate Motif [(R/K)XPSX(R/K)] MultiMab^TM^ (#6967), E-Cadherin (#14472), c-Myc (#13987), p38 MAPK (#8690), and Histone H3 (#4499); from Proteintech, Cofilin 1 (10960-1-AP). Secondary antibodies (HRP-conjugated) were from Santa Cruz Biotechnology (1:2000 dilution) (goat anti-mouse IgG-HRP, sc-2005; goat anti-rabbit IgG-HRP, sc-2004), or Bethyl Laboratories (1:5000 dilution) (mouse IgG-heavy and light chain antibody, A90-116P; rabbit IgG-heavy and light chain antibody, A120-101P).

### 4.5. Bioinformatics Analysis

Deseq2 (1.22.1) was used to perform a differential gene expression analysis on raw RNA-seq counts [32]. Python 3.6.5 and R 3.5.1 were used for statistical analyses. RNA-seq data that support the findings of this study are available from EF upon reasonable request.

The datasets included in this study were downloaded from NCBI’s Gene Expression Omnibus (GEO) (accession numbers: GSE41313, GSE9691, GSE58252, GSE24202) and analysed by GEO2R. Raw data were then exported and graphed in GraphPad PRISM software (version 6). Statistical tests include unpaired two-tailed Student’s t-test, and one-way ANOVA. Comparison of *CA12* expression level in human samples was performed using GEPIA (http://gepia.cancer-pku.cn/index.html). GEPIA is an interactive web server for analyzing the RNA sequencing expression data of 9736 tumors and 8587 normal samples from the TCGA and the GTEx projects [33]. GOBO was used to evaluate the association between *CA12* mRNA levels and molecular subtypes in a cohort of 1881 BC patients. Two molecular subtype classification methods were used: Hu is based on a 306 gene signature that can distinguish different BC subtypes with distinct patient outcome, while PAM50 relies on a 50 gene signature [34].

## Figures and Tables

**Table 1:** Clinical characteristics of breast cancer patients.

Sample ID	Age	Grade	T at Diagnosis	N at Diagnosis	IHC Subtype
01	72	2	2	2a	LumA
02	60	3	1c	2a	TN
03	72	2	1c	x	TN
04	52	3	1c	0	LumA
05	35	3	1c	1a	TN
06	75	3	2	0	TN
07	79	3	1c	x	LumB HER2+
08	78	2	2	0	LumA
09	74	3	1a	2a	TN
10	60	3	1c	1	LumB HER2+
11	66	2	1b	0	LumB HER2+
12	41	3	2	1a	TN

Immunohistochemistry (IHC), primary tumor (T), regional lymph nodes (N).

## Supplementary Materials

The following are available online at https://www.mdpi.com/1422-0067/21/3/715/s1.
