# Supplementary figures and images for: Carbonic Anhydrase XII Expression Is Modulated during Epithelial Mesenchymal Transition and Regulated through Protein Kinase C Signaling
Source: Int J Mol Sci. 2020 Jan 22;21(3):715. doi: 10.3390/ijms21030715 (PMC7037142; doi:10.3390/ijms21030715)

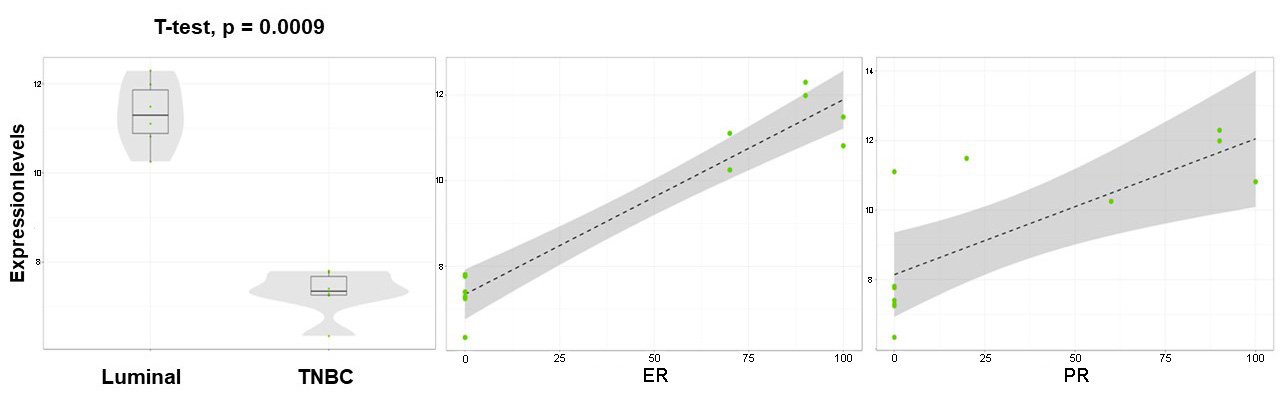

Supplement: Supplementary file 1 [file ijms-21-00715-s001.zip › Suppl. Figure 1 revised.jpg]
